# Maximizing the Potential of Attractive Targeted Sugar Baits (ATSBs) for Integrated Vector Management

**DOI:** 10.3390/insects14070585

**Published:** 2023-06-28

**Authors:** Teresia Muthoni Njoroge, Majidah Hamid-Adiamoh, Molly Duman-Scheel

**Affiliations:** 1 Department of Medical and Molecular Genetics, Indiana University School of Medicine, Raclin-Carmichael Hall, 1234 Notre Dame Ave., South Bend, IN 46617, USA; tenjorog@iu.edu (T.M.N.); mhamidad@iu.edu (M.H.-A.); 2 Eck Institute for Global Health, The University of Notre Dame, Notre Dame, South Bend, IN 46556, USA

**Keywords:** *Anopheles*, *Aedes*, *Culex*, sugar feeding, mosquito control, malaria, insecticide

## Abstract

**Simple Summary:**

Current mosquito control efforts are insufficient for preventing mosquito-borne illnesses. Attractive targeted sugar bait (ATSB) technology is an emerging mosquito control method that involves luring mosquitoes to feed on a sugar suspension containing a poison. Here, we comprehensively review the existing literature to evaluate the potential utility of ATSBs for mosquito control. We highlight milestones in the development of ATSBs, focusing on the selection of toxic ingredients and attractive components, methods of deployment, and efficacy studies. We discuss the potential utilization of ATSBs in combination with other control technologies and identify existing gaps in the ongoing development of this promising technology. We conclude that the deployment of ATSBs in integrated mosquito control programs will help address mosquito control challenges and prevent diseases that result from pathogens transmitted by mosquitoes.

**Abstract:**

Due to the limitations of the human therapeutics and vaccines available to treat and prevent mosquito-borne diseases, the primary strategy for disease mitigation is through vector control. However, the current tools and approaches used for mosquito control have proven insufficient to prevent malaria and arboviral infections, such as dengue, Zika, and lymphatic filariasis, and hence, these diseases remain a global public health threat. The proven ability of mosquito vectors to adapt to various control strategies through insecticide resistance, invasive potential, and behavioral changes from indoor to outdoor biting, combined with human failures to comply with vector control requirements, challenge sustained malaria and arboviral disease control worldwide. To address these concerns, increased efforts to explore more varied and integrated control strategies have emerged. These include approaches that involve the behavioral management of vectors. Attractive targeted sugar baits (ATSBs) are a vector control approach that manipulates and exploits mosquito sugar-feeding behavior to deploy insecticides. Although traditional approaches have been effective in controlling malaria vectors indoors, preventing mosquito bites outdoors and around human dwellings is challenging. ATSBs, which can be used to curb outdoor biting mosquitoes, have the potential to reduce mosquito densities and clinical malaria incidence when used in conjunction with existing vector control strategies. This review examines the available literature regarding the utility of ATSBs for mosquito control, providing an overview of ATSB active ingredients (toxicants), attractants, modes of deployment, target organisms, and the potential for integrating ATSBs with existing vector control interventions.

## 1. Introduction and Historical Perspective of Insect Baiting

Vector-borne diseases pose a major economic and public health burden globally, with mosquito-borne diseases causing more than 700,000 deaths annually [1]. With the rapid emergence and increased prevalence of mosquito-borne diseases, mosquito control is presently the most effective strategy for disease mitigation. Various mosquito control methods are presently employed, including community-based operations for larval source reduction through the removal of aquatic breeding sites, biological control through the use of natural predators, microorganisms, or modified mosquitoes, and chemical control through pesticide applications. For many decades, chemical-based insecticides have been the backbone of mosquito control programs, which rely on insecticide spraying, impregnated bed nets, fogging, and larviciding [2]. However, the effectiveness of these interventions continues to decline due to the rapid spread of insecticide resistance to all four major classes of synthetic chemicals in mosquito populations [3], and non-target effects, in addition to public and environmental safety, are of concern [4]. Therefore, the current toolbox for mosquito control is insufficient to support ongoing efforts toward disease mitigation, necessitating the discovery and implementation of new methods for integrated mosquito management that are effective and environmentally safe. Several promising new strategies under consideration include the sterile insect technique (SIT), gene drives, the incompatible insect technique (IIT), the release of insects carrying a dominant lethal gene (RIDL) [5,6], and ATSB technology [7]. ATSBs, a new form of insect baiting, employ an attract-and-kill strategy that exploits the sugar-feeding behavior of adult mosquitoes.

Insect baiting is a medieval concept that is used to kill or dissuade pests of agricultural, urban, and medical importance. The concept dates back to 77 A.D. when Pliny the Elder, a Roman naturalist, hung a fish on a tree next to vegetation to repel ants, as described in *Historia Naturalis* [8]. He also used plant-based sugar lures to target insects. In the 1920s, toxic sugar baits (TSBs) containing arsenic were used to control termites in Australia, and the usefulness of the baits for termite control was again documented in the 1930s when a sugar solution containing sodium arsenite was found to kill harvester termites [9]. In the 1970s, wooden blocks treated with mirex, an organochloride, were used as baits to control termite attacks on southern pines in the USA [10]. In the 1950s, insecticides mixed with food and protein hydrolysate as the attractant were used on a large scale to control the Mediterranean fruit fly in Hawaii [11]. This baiting technique has been modified several times and is applied to control fruit flies around the world. By the mid-19th century, insect baits targeting urban pests were prepared by mixing an insecticide with regular food [12]. In the 1960s, various baits, including Kepone mixed with peanut butter as a food bait, as well as mirex mixed with soy corncob grit, were produced and widely used for fire ant control [11]. In 1965, the first mosquito toxic sugar bait formulation was pioneered in the laboratory by Lea, who was targeting *Aedes aegypti* on a TSB-treated paper. The sugar bait included malathion as a toxicant and a 20% sugar solution as a feeding stimulant [13]. The invention and use of the metabolic inhibitor hydramethylnon, which was included in food baits for cockroaches and ant control in the 1990s, paved the way for the development of food baits used for insect control. Since then, more refined and sophisticated bait formulations have been developed on a large scale and commercialized, consequently helping to revolutionize insect baiting as part of integrated pest management (IPM) on a worldwide scale. Furthermore, the invention of slow-acting and highly effective insecticides that are not topical poisons has tremendously improved baiting for insect pest control [14].

The evaluation of TSBs composed of a toxicant and a sugar bait led to an understanding of the shortcomings of this method for mosquito control. The limited attractiveness of the sugars mixed with the toxicants, which were not as attractive as natural sugar sources, diminished the efficacy of this intervention [15,16,17]. To address this, alternative mosquito attractants were explored as possible components of the TSBs. The concept of attractive baits was first explored in 1910 when plant-based attractants were discovered for the control of cabbage butterfly larvae against mustard plants [8]. In the 1920s, the potential of several attractive aromatic compounds to dissuade orchard insects from visiting plants, their food source, was explored [8]. These evaluations and the discovery of compounds with attractive scents revolutionized insect control approaches through attractive baiting. ATSBs have been explored extensively in tropical regions where vector-borne diseases are endemic. Sugar feeding behavior has been observed in mosquitoes [18,19,20], sandflies [21,22,23], and black flies [23], demonstrating that these insects utilize sugar as an energy source. Plant sugar is a critical dietary requirement for most adult mosquitoes [18]. Soon after emergence, both males and females seek sugar as an energy source for mating [24] and continue to utilize the carbohydrate source throughout their lifespans. The males exclusively feed on sugars throughout their lifetime while the females periodically sugar feed in between blood feeding cycles to obtain energy reserves. Sugar meals enhance the fitness and reproductive success of both sexes [25]. Since 1965, there has been tremendous development in the use of sugar baiting for mosquito control ranging from TSBs to the current ATSB regimens that employ a commercially formulated attractant. The ATSB solution consists of a scented compound that is attractive to the target vector, a sugar component that encourages feeding (feeding stimulant), and an oral toxicant (insecticide) designed to induce mosquito mortality/morbidity upon ingestion of the solution [20]. 

Here, we provide an overview of ATSB technology that focuses on active ingredients and attractants used in ATSBs, various modes of ATSB deployment, a summary of efficacy studies, and the promise, challenges, and future work required for achieving the long-term goal of integrating this promising new technology with existing vector control interventions.

## 2. Active Ingredients Used in ATSBs

Active ingredients are critical ATSB components that make sugar meals toxic and lethal to target mosquitoes. To date, several toxic compounds, including chemicals, biopesticides, and plant products, have been evaluated, as reviewed in detail by Fiorenzano et al. [7]. Chemical-based toxins in ATSBs have mainly included different classes of insecticides that are approved for vector control [26], including bendiocarb, carbamates, neonicotinoids, organophosphates, pyrethroids, pyrroles, and fipronil [27,28,29,30]. Furthermore, other chemicals, such as boric acids [31,32,33], dinotefuran [27], eugenol [34], ivermectin [35], and spinosad [36], as well as several others [7], have been utilized. Chemical insecticides have been shown to be highly potent and cause significant mortality in *Aedes*, *Anopheles*, and *Culex* mosquitoes [7,28,37]. Unfortunately, chemical insecticides can be toxic to mammals and non-target insects [38], hence, efforts to optimize ATSBs with other active ingredients were accelerated [7]. The use of bacterial and fungal spores as active ingredients in ATSBs was evaluated using spores from *Bacillus sphaericus* and *B. thuringiensis israelensis* (Bti) [39,40,41], *Pseudomonas* species [42] and the fungal strain *Metarhizium anisopliae* [43], which successfully killed adults and larvae of *Anopheles*, *Aedes*, and *Culex* mosquitoes, as well as the sand fly [44]. Furthermore, ATSB, boric acid or eugenol sugar baits formulated with pyriproxyfen, an insect growth regulator, significantly inhibited adult *Aedes albopictus* emergence in a simulated rain-wash experiment. Treatments containing pyriproxyfen applied to plants were transferred by adults to larval habitats, in which the insecticide induced up to 100% adult emergence inhibition [45,46].

ATSBs prepared with naturally derived active ingredients, which were expected to be environmentally friendly and safe, have also been explored. These include plant-based products, such as eugenol, garlic oil (microencapsulated), and sodium ascorbate. When incorporated into sugar baits, these active ingredients have been shown to kill mosquitoes and are non-toxic to non-target insects [47,48,49]. It was suggested that the use of plant-based ATSBs be prioritized to minimize the toxic effects of ATSBs on non-target insects [38]. Nonetheless, the use of these ingredients is limited by their relatively short half-life, which is reduced with respect to chemical insecticides. These insecticides are often relatively more expensive and challenging to synthesize [50]. 

**Figure 1:**
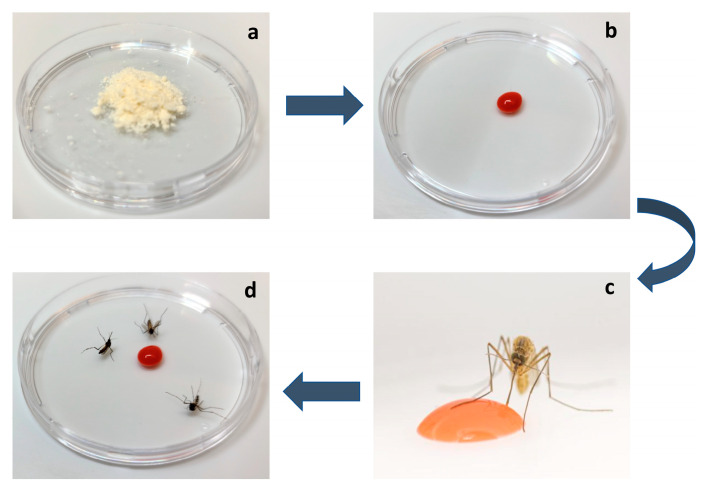
RNAi-Yeast ATSB Technology. Dried heat-inactivated RNAi yeast (**a**) is prepared as an ATSB suspension (**b**) that is fed to mosquitoes (**c**) which die after consuming it (**d**). A *Culex quinquefasciatus* female feeding on the ATSB suspension is shown in (**c**).

RNA interference (RNAi) technology has advanced as a useful research tool that could potentially be applied in operational vector control strategies [51,52]. Interfering RNA insecticides utilize the innate RNAi mechanism in mosquitoes for the species-specific RNAi-mediated silencing of essential mosquito genes, resulting in mortality. Several siRNAs targeting essential mosquito genes have been screened and tested as larvicides and adulticides. Short hairpin RNAs (shRNAs) corresponding to the siRNA sequences can be produced and delivered using modified yeast (*Saccharomyces cerevisiae*) [53] to target *Aedes*, *Anopheles*, and *Culex* mosquitoes [54,55,56,57]. The bioengineered yeasts, which are heat-inactivated prior to deployment, have been incorporated as toxicants in ATSBs targeting adult mosquitoes [54,55,56,57]. These were shown to effectively kill mosquitoes in the laboratory [54,55,56,57] (Figure 1). Moreover, double-stranded (ds) RNAs matching essential *An. gambiae* genes were successfully expressed in *Escherichia coli*. The heat-inactivated bacteria can also be delivered orally to adult mosquitoes, resulting in defects in salivary gland morphology [58].

Significant efforts are underway to further evaluate yeast interfering RNA-based ATSBs under semi-field conditions, and these studies should be followed by large-scale field trials that assess entomological endpoints. Yeast RNAi technology allows for the development of safe and effective mosquito control insecticides as an addition to the existing mosquito control toolbox. *S. cerevisiae*, commonly known as baker’s yeast, a delivery system for interfering RNA insecticides, is non-toxic to humans and is used globally in the food and beverage industry. Yeast has a strong odor and is highly attractive to both larvae and adult mosquitoes, and the insecticidal properties of yeast are preserved when inactivated, permitting the use of dead microbes for mosquito control [59]. Additionally, *S. cerevisiae* is genetically tractable, enabling the generation of unique yeast RNAi insecticide strains that target different essential genes in mosquitoes, providing an avenue for insecticide resistance management through interfering RNA insecticide rotation [59,60]. The propagation of interfering RNAs through yeast culturing allows for scaled insecticide production, reducing the cost of mosquito control products. The species-specific yeast interfering RNA insecticides are designed to only match mosquito genes and do not affect non-targeted arthropods including *Apis mellifera* (honeybees), *Drosophila melanogaster* (fruit flies), *Tribolium castaneum* (red flour beetle), and *Oncopeltus fasciatus* (milkweed bugs) [54,55,56,57]. Moreover, the potential for delivering yeast interfering RNA insecticide-based ATSBs through a porous black plastic membrane [30] could serve to protect the sugar bait from non-target organisms and adverse environmental conditions, preserving its shelf life. In addition to combating insecticide resistance, yeast interfering RNA-based ATSBs are promising candidates for integrated mosquito management both indoors and outdoors.

In summary, although a variety of different broad-based toxins have historically been deployed as ATSBs, recent efforts have focused on the identification of more selective toxins, including RNAi-based insecticides, that selectively kill mosquitoes. Such efforts aim to increase the environmental safety of this intervention.

## 3. Attractants Incorporated into TSBs

The discovery of attractive aromatic compounds engineered to lure insect pests away from their natural food sources [8] has increased the efficacy of TSBs. Plant-based attractants are the oldest form of aromatic compounds to be incorporated into baits for the control of insects that damage crops. At the end of the 19th century, attractants were mixed with baits to prevent insect pests from attacking crops. In 1910, plant-based attractants were used to dissuade cabbage butterfly larvae from visiting mustard plants [8]. By the 1920s, dozens of attractive aromatic compounds were used to dissuade orchard insects from visiting plant food sources [8]. 

Initial studies evaluating ATSBs for mosquito control used local plants as attractants by spraying the vegetation with a colored sugar bait [19]. The application of TSBs on flowering plants was, however, not practical because non-target arthropods were also attracted and killed. To address this, the ATSB studies explored overripe fruits as attractants, adding sucrose as a feeding stimulant in the bait. This allowed for the application of fruit-based ATSB solutions on non-flowering plants to discourage non-target organisms from feeding on the baits [7]. Since 2008, several fruit-based sugar sources have been explored in various mosquito species both in the laboratory and field studies, with the addition of attractants in ATSBs resulting in up to 97% mosquito mortality [7,61]. Aside from plant-based attractants, CO_2_ presented with TSBs was found to be effective against *A. aegypti* and *A. taeniorhynchus* in semi-field and field studies [62]. However, an attempt to incorporate human host kairomones L-lactic and 1-octen-3-ol, which are known to attract mosquitoes to fruit-based sugar baits, did not enhance mosquito attraction and efficacy of ATSBs in laboratory or semi-field studies [63].

The use of flowering plants, fruits and sugar sources as attractants in ATSBs is challenging for the long term, given the lack of residual activity and potential negative impacts on non-target organisms. This limitation paves the way for the development of more stable commercial attractant formulations that could be delivered in a manner that is less toxic to non-targets. The commercial ATSB attractant developed by Westham Co., Dallas, TX, USA has been evaluated with multiple active ingredients including dinotefuran, eugenol, and garlic oil, yielding varying levels of mosquito mortality ranging from 62 to 98% in *Anopheles, Aedes*, and *Culex* mosquitoes [34,47,49,64]. 

In summary, the use of attractants that can effectively lure mosquitoes away from feeding on natural sugar sources is key to the successful deployment of ATSBs. However, the field is evolving away from the combined deployment of broad-based attractants with broad-based toxins, which could have negative impacts on non-target species. The identification and use of selective attractants will limit the impacts of broad-based toxins on non-targets; thereby, making ATSBs a more environmentally friendly mosquito control intervention.

## 4. Methods of ATSB Deployment

Laboratory and field trials have successfully tested ATSB effectiveness by spraying the toxic bait on vegetation or by delivering it in mounted bait stations [27,29,30,55,64]. Vegetation spraying is mainly performed to target exophilic vectors outdoors [19,49]; however, several studies have also applied sprays to indoor vegetation [7,31]. Trials using the vegetation spraying method on flowering (attractive) and non-flowering plants (non-attractive) have recorded similar mosquito mortality rates [34,47,63]. A drawback associated with this method is the indirect impact on non-target insects [49]. Studies [49,65] have suggested that spraying ATSBs on flowering vegetation could attract more non-target insects compared to non-flowering vegetation. In addition, the use of low-risk active ingredients minimized the impact on non-target insects [47,65]. Nonetheless, research efforts to identify safe, environmentally friendly ATSBs should be intensified.

Bait stations are deployable both indoors and outdoors, offering the potential for broader impacts on endophilic and exophilic vectors [30,64]. Several prototypes and designs of ATSB bait stations have been developed to achieve a functional platform that can be positioned indoors and/or outdoors, and which is effectively accessible to vectors to feed and rest, while also preventing access to non-target insects. Some of these designs have used movable frames on which ATSB-impregnated cotton balls, cotton towels, cotton wicks, membranes, and tubes were mounted, and all have been effective for ATSB delivery [27,33,58,65]. The bait station manufactured by Westham Innovations LTD (Israel) has been extensively evaluated in field studies in Africa [27,66,67]. It is made of a rectangular plastic frame for mounting ATSB solutions and covered with a protective black porous plastic membrane that selectively permits mosquito probing [28,31], protecting the sugar bait from non-target organisms and adverse environmental conditions while also preserving shelf life. Such efforts to protect non-targeted organisms led to re-naming this intervention attractive *targeted*, rather than *toxic*, sugar baits [67], as current efforts prioritize targeting mosquitoes while minimizing impacts on non-targets. Likewise, such terminology is appropriate for RNAi-based ATSB applications [50,51] and any similar efforts that prioritize limiting the impacts of sugar bait technology to the intended pests.

## 5. ATSB Efficacy Studies

The potential for ATSBs to reduce mosquito densities has been demonstrated through efficacy studies [27,47,64]. To assess the impacts of the differences in spatial and environmental conditions, as well as the genetic diversity of the vector populations on ATSB efficacy, studies have been conducted in Africa, the Middle East (Israel), and the United States (Florida) [7,27,34]. The studies in Africa evaluated ATSB efficacy on the *A. gambiae* complex in a tropical environment abundant in alternative sugar sources that can distract vectors from feeding on ATSBs [30,68,69]. Similar studies in Israel assessed ATSB efficacy in an arid and sub-arid setting with poor alternative sugar sources, evaluating several vector species including *Anopheles claviger, A. gambiae, Anopheles sergentii, Aedes caspius*, and *Culex pipiens* [17,41]. The trials conducted in Florida enabled the assessment of ATSB efficacy in sub-tropical ecosystems with intermediate sugar source availability in which *Aedes* and *Culex* are predominantly found [7,34]. The observed variations in the efficacy and responses in the available ATSB studies are attributed to weather and environmental factors that include humidity and temperature [29,65], plant species and flowering state [36,37,39], and the type of species and physiological states of the mosquitoes [33,70,71]. All studies demonstrated good ATSB efficacy regardless of the ecological settings and vector genetic diversity, validating the potential for ATSBs as a novel vector control tool [7].

**Figure 2:**
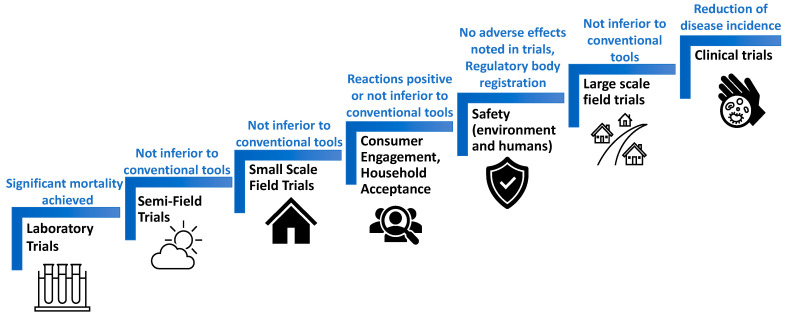
Development and evaluation plan for safe, effective, and stakeholder-accepted ATSB technology. The ideal outcomes (blue) at each stage of the process (black) are noted.

As ATSBs are currently under development (Figure 2), most efficacy studies have measured the efficiency of this intervention using key entomological indices that evaluate toxicity to mosquitoes. For laboratory-based studies, direct mosquito mortality and feeding rates are measured as primary endpoints [31,35,55]. The feeding rate can be assessed when a food dye marker is incorporated into ATSB suspensions. A few studies [7,31] also compared the feeding rates between male and female mosquitoes to determine ATSB efficacy in both mosquito populations. Semi-field and field trials that measure entomological outcomes have included the mortality rate, feeding rate, vector density (vector abundance), vector composition, vector parity, human biting rate (HBR), sporozoite infection rates, the density of older female vectors, vector longevity, entomological inoculation rates (EIR), and several other factors [27,29,30,65,66]. Most of these studies collect baseline data to compare with post-ATSB intervention data.

Measuring the epidemiological outcomes is critical to assessing ATSB efficacy (Figure 2). This involves measuring the impact of the strategy to reduce disease morbidity and mortality. A Phase 3 clinical trial [67] is currently underway in three African countries and aims to determine the ATSB impact on malaria when combined with long-lasting insecticidal nets (LLINs) and indoor residual spraying (IRS). The primary endpoint to be measured is the reduction in clinical malaria incidence and parasite prevalence by at least 30%. Moreover, recently, a modeling study [37] measured the vectorial capacity and vectorial competence of *A. albopictus* as epidemiological indices to predict the potential for ATSBs to reduce dengue virus transmission; however, this has not yet been assessed in the field. Demonstrations of the ability of ATSBs to reduce the incidences of mosquito-borne diseases, combined with the detection of significant impacts on entomological endpoints, will support future registry applications.

## 6. Incorporating ATSBs in Integrated Vector Management (IVM)

The WHO IVM strategy encourages the discovery and application of additional vector control interventions parallel to existing strategies for reducing the transmission of vector-borne diseases [72,73]. Combining several approaches may allow for the control and prevention of several vectors and diseases simultaneously while helping to ensure vector control sustainability. Integrated approaches become necessary because of the adaptability of mosquitoes to commonly used control methods. Integrating ATSBs, a strategy that manipulates and exploits mosquito sugar feeding behavior, would present a beneficial addition to current vector control interventions.

The majority of competent vectors of key mosquito-transmitted pathogens are strongly associated with humans and are commonly encountered in the home environment, both indoors and outdoors [74]; this includes malaria, dengue, Zika, and lymphatic filariasis mosquito vectors. Several vector control interventions are employed in the peri-domestic environment to prevent biting by the disease vectors. These include conventional LLINs and IRS that utilize pyrethroids and larval source reduction to reduce malaria parasite and arbovirus transmission [75]. Other commonly used approaches to prevent mosquito bites in the home environment include lethal baited traps, mosquito-proof housing, and the use of topical or spatial repellents [76]. Although conventional LLINs and IRS interventions have been effective in malaria prevention, additional approaches are needed in the home environment to address changes in mosquito behavior (from indoor to outdoor biting) [77,78] and widespread resistance to commonly used pyrethroids by mosquito populations [3], as well as human activities that are primarily outdoors, increasing bite exposure. 

Beyond lab and field studies, there has been a significant enhancement of ATSB utility for mosquito control in the field. The current ongoing large-scale community-based ATSB trials in multiple locations in sub-Saharan Africa [67], the hotbed of malaria transmission, could prove the efficacy of ATSBs as effective and innovative interventions for mosquito control both indoors and outdoors. Apart from larval source reduction, an approach that has not widely been used to prevent malaria [75], current mosquito control methods are focused indoors using LLINs and IRS interventions to reduce the spread of malaria. Both male and female mosquitoes resting indoors and outdoors in the home environment acquire sugar meals from natural plant sources [79]. ATSBs have the potential to control both indoor and outdoor populations, and this is particularly important to reduce the residual transmission that results from outdoor biting, a new behavior exhibited by mosquitoes which renders traditional methods, such as LLINs and IRS, less effective [77,78]. Targeting the control of malaria in Africa, studies have assessed ATSB efficacy when deployed as an indoor and/or outdoor strategy, in combination with existing vector control strategies. A previous study in Mali [34] tested ATSBs placed indoors along with existing indoor-based LLINs. The study first recorded high vector feeding rates from ASB bait stations marked with food dye. Subsequently, the investigators observed a significant reduction in the house entry of both male and female vectors and reduced densities of older female mosquitoes. Another study in Mali [31] tested ATSBs positioned outdoors in study villages where LLINs were implemented indoors. The study observed similar results of high vector feeding rates, a reduction in vector density, and decreased malaria transmission indices. Research studies assessing the utility of ATSBs for the control of arboviral vectors that breed in both natural and artificial containers are limited [80], and this calls for renewed efforts to evaluate the vector control potential of this intervention.

With regard to insecticide resistance management, ATSB insecticides have proven effective in multiple settings. Studies with indoor ATSBs in Tanzania [29] and Cote d’Ivoire [68] observed high efficacy against pyrethroid-resistant malaria vectors, indicating an added potential for ATSBs to control resistant malaria vectors. Evidence for the control of arboviral diseases was also documented [29,47,49], with dramatic decreases in the population of *Aedes* and *Culex* arboviral vectors following the ingestion of ATSBs. The ability to control resistant arboviral vectors was also demonstrated in a study conducted in Tanzania [29]. Using data from ATSB efficacy trials, modeling studies [80,81] substantiated the evidence that ATSBs will be effective in preventing malaria, particularly if integrated with existing control strategies. These models further suggested that ATSBs could help address residual malaria transmission. Another study [81] indicated that the efficacy of ATSBs will not be affected by the availability of alternative sugar sources, as previously suggested [82]. Such modeling studies suggest that the pursuit of field trials to assess the entomological and epidemiological impacts of ATSBs deployed as components of integrated mosquito and disease control programs will be beneficial.

## 7. Challenges and Future Directions

Addressing widespread insecticide resistance by mosquito populations in conjunction with vector species’ invasions into new territories will require new means of mosquito control. For example, the recent invasion of *Anopheles stephensi* in Africa calls for heightened surveillance efforts and the discovery of novel and suitable mosquito control interventions. Numerous research efforts provide evidence that ATSBs have the potential to reduce mosquito populations. Further to this development, ATSBs offer significant promise as innovative tools for integrative mosquito management in the home environment, which is in line with IVM, a WHO strategy [72,73] for maximizing vector control to reduce the transmission of vector-borne diseases. Although conventional LLINs and IRS have been effective for the control of malaria vectors indoors, a large gap exists in settings with high disease endemicity for the control of mosquitoes that bite outside of homes [73]. ATSBs provide a perfect addition to existing interventions for the control of outdoor biting mosquitoes. Recent developments provide evidence that innovative tools have the potential to significantly reduce mosquito densities and clinical malaria incidence in multiple locations in sub-Saharan Africa [30,67], and studies at additional geographical locations should be performed, as this will facilitate the optimization of ATSB strategies in a variety of different environments. Furthermore, the combined control of arboviral vectors through larval control in combination with other interventions targeting adults will facilitate sustained arboviral disease control. Most large-scale evaluations of ATSB utility have focused on malaria vectors. Very few ATSB studies on *Aedes* and *Culex* vectors have been conducted in the field to date, and this calls for large-scale trials to be focused on key arboviral vectors in tropical regions that are endemic to arboviral infections. To date, ATSB studies on *Aedes* vectors have mostly been based in the state of Florida, USA [17,34,63,64,71], and a recent trial was also conducted in rural areas of Mali [83]. Given that these studies demonstrate that ATSBs can be applied for arboviral mosquito control, there is a need to identify the most effective ATSB attractants for these species and to reproduce these studies in urban settings that harbor abundant human hosts, particularly given that *A. aegypti* and *A. albopictus* females are known to blood feed several times in a single gonotrophic cycle [84], which increases the risk of disease transmission.

Current ATSB trials are evaluating the deployment of ATSB insecticides in bait stations that deliver the bait through a porous black plastic membrane [31], which protects it from non-target organisms and adverse environmental conditions, preserving the shelf life of the bait. These bait stations are easy to install outside human dwellings, making the technology easy to adopt and use in targeted communities. Furthermore, the ATSB formulations target multiple mosquito species and have extended residual activities [67], which may make them cost-effective. To maximize the effectiveness of ATSBs for integrated mosquito management, several improvements are recommended. Since the ATSBs must outcompete natural plant sugar sources, there is a need to maximize the behavioral responses of various mosquitoes through the identification of baits that are optimally attractive to each mosquito species. This will maximize mosquito attraction toward the sugar baits, improving their effectiveness. To prevent incidences of ATSB insecticide resistance, researchers should explore combining active ingredients with multiple modes of action against the mosquito vectors. On matters pertaining to the environmental safety of ATSB insecticides, developers should explore innovative insecticidal ingredients that are safe and non-toxic to non-target organisms. The novel yeast RNAi-ATSBs [54,55,56,57], for example, were designed to specifically target disease vector species and fit these requirements. Moreover, the yeast is highly attractive to mosquitoes, which may permit the development of highly attractive baits with a toxin that selectively targets mosquitoes. Ongoing semi-field trials are evaluating yeast that has been suspended in sugar bait and delivered to mosquitoes in sachet bait stations. These trials will examine the residual activity of the interfering RNA when deployed using sugar baits in the field, a key component of evaluating the overall efficacy of this new technology.

Furthermore, although ATSB insecticides target adult mosquitoes, attempts to simultaneously kill immature stages of *Anopheles, Aedes,* and *Culex* mosquitoes using ATSBs formulated with biopesticides [39,40,41,42,43] and pyriproxyfen [45,46] have been successful in the laboratory; thus, more effort should be focused on field studies to better assess the efficacy of this ATSB-deployed technology for targeting container breeding mosquitoes. Modeling studies have also shown that optimizing the deployment of ATSB bait stations to target resting sites, sugar sources, and larval habitats, could significantly improve the effectiveness of these mosquito control interventions [81].

Additionally, to promote the adoption of ATSBs for commercial use, the developers and mosquito control programs should continuously pursue community engagement regarding the use of ATSBs for mosquito control in the home environment (Figure 2). This will help to ensure acceptance and adoption of the technology by the targeted end-users. Although developments concerning the commercial use of sugar baiting for mosquito control have taken several decades to be realized, significant strides toward the registry and adoption of ATSBs for mosquito control are occurring, as evidenced by the recently completed large-scale ATSB trial in Mali [30] and the ongoing Phase 3 trials in Mali, Zambia, and Kenya [67]. These significant developments have secured ATSBs a place on the list of mosquito vector control interventions that are currently under review by the vector control advisory group (VCAG) of the WHO, which will determine their public health significance for vector control [85].

## 8. Conclusions

ATSBs are a promising mosquito control intervention that can be designed to have minimal impacts on the environment. Continuing efforts to identify the most effective and stakeholder-accepted combinations of baits, toxins, and deployment strategies for the selective targeting of *Aedes, Anopheles,* and *Culex* mosquitoes are expected to further enhance existing ATSB strategies. It is anticipated that this promising new mosquito control intervention will seamlessly combine with existing mosquito management strategies to facilitate the advancement of sustainable mosquito-borne disease elimination.

## Data Availability

Data sharing is not applicable to this article.
